# Beyond DNA: An Integrated and Functional Approach for Classifying Germline Variants in Breast Cancer Genes

**DOI:** 10.1155/2016/2469523

**Published:** 2016-10-16

**Authors:** T. Pesaran, R. Karam, R. Huether, S. Li, S. Farber-Katz, A. Chamberlin, H. Chong, H. LaDuca, A. Elliott

**Affiliations:** Ambry Genetics Corp., 15 Argonaut, Aliso Viejo, CA 92656, USA

## Abstract

Genetic testing for hereditary breast cancer is an integral part of individualized care in the new era of precision medicine. The accuracy of an assay is reliant on not only the technology and bioinformatics analysis utilized but also the experience and infrastructure required to correctly classify genetic variants as disease-causing. Interpreting the clinical significance of germline variants identified by hereditary cancer testing is complex and has a significant impact on the management of patients who are at increased cancer risk. In this review we give an overview of our clinical laboratory's integrated approach to variant assessment. We discuss some of the nuances that should be considered in the assessment of genomic variants. In addition, we highlight lines of evidence such as functional assays and structural analysis that can be useful in the assessment of rare and complex variants.

## 1. Introduction

The landscape of genetic testing for hereditary breast cancer susceptibility has changed drastically with the application of massively parallel sequencing based tests in clinical diagnostics. Clinical genomic laboratories are performing an increasing number of massively parallel sequencing assays for cancer predisposition genes [1], which has led to an intensified application of these assays in clinical and research settings [2]. Breast cancer gene panels and exome sequencing generate vast amounts of genetic alteration data, thereby presenting a significant challenge to determine which variants are responsible for the disease or phenotype. Multigene breast cancer panels in particular have gained in popularity over the past few years and are now routinely ordered by genetics, oncology, and breast surgical clinics. These tests allow for simultaneous analysis of numerous cancer genes that, when mutated, can have a significant impact on cancer risk stratification and management [3]. A major component of clinical molecular diagnostic testing is accurate assessment and interpretation of genetic variants.

Ambry Genetics' BreastNext Cancer panel analyzes 17 genes (*ATM, BARD1, BRCA1, BRCA2, BRIP1, CDH1, CHEK2, MRE11A, MUTYH, NBN, NF1, PALB2, PTEN, RAD50, RAD51C, RAD51D*, and* TP53*) by massively parallel sequencing of all coding exons and a minimum of 5 base pairs into the flanking 5′ and 3′ ends of all introns and untranslated regions. In addition, clinically significant intronic mutations beyond 5 base pairs and the promoter region of* PTEN* (c.-1300 to c.-745) are always sequenced and reported. Sequencing is conducted on the Illumina HiSeq2500 or NextSeq using 150 bp paired-end conditions as described in the manufacturer's standard workflow (Illumina). After initial data processing, all clinical samples had to pass minimum thresholds to be included in the analysis. The three parameters were as follows: mean base calling quality score is greater than 30, the percentage of passes that reached over 30 had to be 75% overall, and the percentage of perfectly matched indexes needed to be greater than 85%. For each gene, a minimum coverage of 20x is required for candidate variants to be called.

In an effort to help standardize the interpretation and reporting of genetic testing results, organizations such as the American College of Medical Genetics and Genomics (ACMG), Association for Molecular Pathology (AMP), and the International Agency for Research and Cancer (IARC) have proposed criteria for the interpretation and reporting of sequence variants [7–9]. These criteria weigh multiple lines of evidence to categorize variants under a five-tier classification algorithm using terms such as pathogenic (P), variant, likely pathogenic (VLP), variant of unknown significance (VUS), variant, likely benign (VLB), and benign (B) to indicate the likelihood of association with disease. Per ACMG guidelines, the term “likely” refers to a classification tier that equates to a >90% likelihood of a variant being disease-causing or benign [7, 8]. Recently the clinical utility of the ACMG guidelines was demonstrated in a cohort of individuals undergoing sequencing for inherited cancer risk [10].

While the ACMG guidelines provide a basic framework for variant assessment, gene and syndrome-specific factors such as penetrance, prevalence, inheritance pattern, disease mechanism, and protein structure and function need to be considered. In addition, when considering the phenotype of the patients in which a variant is identified, one must take into account the prevalence of the disease and how the patients are ascertained to account for potential phenocopies. For example, many genes on hereditary breast cancer panels are considered to be moderate penetrance and are associated with a 2- to 5-fold increased breast cancer risk. Given the relatively high prevalence of breast cancer (1/8 women in the US), traditional segregation methods are confounded by phenocopies and are even more difficult to employ with genes that have reduced penetrance. These confounders indicate that these genes require large numbers of segregation events to provide meaningful results. Consideration should also be given to gene-specific factors such as frequency of germline and somatic* de novo* alterations, additional tests in tumors such as loss of heterozygosity studies, variation in nonsense-mediated decay, and alternate splicing. For example, in genes such as* TP53* and* PTEN*, germline* de novo* variants are known to be a relatively common cause of disease [11, 12]. However, with breast cancer genes such as* ATM*,* CHEK2*, and* PALB2*, the* de novo* rate is unknown. This is confounded by the fact that breast cancer is a common disease and one cannot infer if the* de novo* event in these genes directly correlates with disease or occurred by chance. In addition, although somatic* de novo* data is available for some genes [13] its incorporation into germline variant analysis has yet to be standardized and will need to be performed on a gene-by-gene basis.

Consortia such as the Evidence Based Network for the Interpretation of Germline Mutant Alleles (ENIGMA) have demonstrated the power of a collaborative approach to variant assessment and have made great strides in the reclassification of VUS in breast cancer genes as pathogenic or benign. However even these groups are limited by the rate at which data is accumulated. Open-access databases such as ClinVar and the Leiden Open (source) Variant Database (LOVD) can be useful in identifying additional cases or publications related to a variant. These databases have also helped standardize the interpretation of variants between laboratories by identifying discrepancies in classifications. Collaborative efforts by clinical laboratories including Ambry Genetics, GeneDx, University of Chicago, and Laboratory for Molecular Medicine have resulted in the sharing of internal data consisting of segregation and cooccurrences with mutations in the same gene or other genes and* de novo* observations have led to the resolution of 78% of clinically actionable differences (VUS versus VLP/mutation) and 92% of VUS versus likely benign/benign differences (internal data). Despite these efforts, one of the challenges faced by molecular laboratories and clinicians is that many genetic variants are very rare and do not have enough published data to be classified beyond VUS. We present here our laboratory's integrated approach to variant assessment and review tools used to assess the impact of variants on protein function.

## 2. Integrated Approach to Variant Assessment

Ambry Genetics has developed and implemented an integrated approach to variant assessment (Table 1) that encompasses a five-tier variant classification algorithm similar to those presented by ACMG and IARC. Although the foundation of Ambry Genetics' classification algorithm is based on the ACMG guidelines, we have adopted stringent thresholds similar to those proposed by the IARC, where “likely” refers to a >95% confidence of a variant being disease-causing or benign [9]. In this algorithm, both pathogenic and likely pathogenic variants are interpreted as clinically actionable with recommendations for medical management and family member testing.

**Figure 1:**
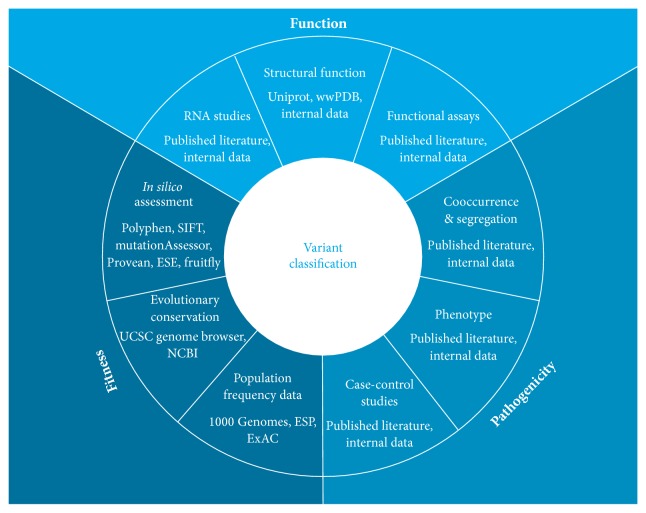
An integrated approach for variant classification. Lines of evidence such as structural function, RNA studies, and functional studies assess the functional impact on the mRNA and protein. Cooccurrence, segregation, case-control studies, and the observed phenotype in variant carriers reflect the pathogenicity of a variant. Population frequency,* in silico* models, and evolutionary conservation assess fitness of the amino acid or nucleotide position.

Ambry Genetics' algorithm incorporates multiple lines of evidence aimed at assessing both the impact of the variant on the protein and the pathogenicity of the variant in relation to a disease phenotype (Figure 1 and Table 2). These lines of evidence are weighted as stand-alone (categories A and F), strong (categories B and D), or supportive (categories C and E) and when combined as described in Table 1, they can lead to a classification of likely benign, benign, likely pathogenic, or pathogenic. When the evidence is limited or conflicting, the variants remain classified as VUS. Lines of evidence such as its location, structure-function, and functional and RNA studies reflect the functional impact on the mRNA or protein. Evolutionary conservation,* in silico* models such as Polyphen and SIFT, and general population frequency reflect fitness, that is, reproductive success and survival as measured by a lack of allelic diversity. The observed phenotype in variant carriers and the cosegregation of the variant with disease and the cooccurrence with other pathogenic variants reflect the pathogenicity of the variant (Figure 1). Some of this evidence is readily available via databases such as allele frequency data in the Exome Aggregation Consortium (ExAC) or the data in published literature [14]. However published literature generally contains data for common variants and the data supporting pathogenicity for rare variants is scarce and frequently only available internally.

For most genes on breast cancer panels, computational data from* in silico* models, evolutionary conservation, and protein structural analysis are readily available. Population frequency data has been accumulating at a fast pace due to major contributions from 1000 Genomes, NHLBI Exome Sequencing Project (ESP), and ExAC. These data have had a significant impact on the identification of benign variants at high frequencies that are too frequent to be pathogenic based on disease incidence alone, particularly for historically understudied ethnic groups. For breast cancer genes, this threshold has been conservatively set at an allele frequency of 1% in large population cohorts if used as a stand-alone line of evidence supporting benign classification (Table 1, category F). Careful consideration of population cohort size is needed to attain a high confidence (lower 95% CI is above 1% with *p* value <0.05) that the frequency is above 1%. For example, with a cohort of 60,000 alleles, an allele frequency of 1.08% is sufficient (lower 95% CI = 1.01%; *p* = 0.0244) whereas for a cohort of 1000 alleles, an allele frequency of 1.70% (lower 95% CI = 1.15%; *p* = 0.013) is needed to be 95% confident; the allele frequency is above 1%.

Although patient phenotype, cooccurrence, and cosegregation data can be found in the published literature, many laboratories also curate internal data for use in variant classification. A patient's clinical and family history can be difficult to use as a line of evidence in a clinical laboratory setting due to ascertainment bias. However, when a variant in a gene associated with a rare disorder (less than 1/2000) is identified in multiple individuals meeting classic clinical criteria and never in large control populations or population cohorts these data can be used as evidence towards pathogenicity. This is most informative in patients who have undergone genetic testing on large multigene panel tests in which all the known genes associated with a disorder have been ruled out. However, when defining classic clinical criteria we use very strict guidelines and exclude common diseases such as breast cancer. For example, when assessing a* TP53* variant, the phenotype is considered strong if the patient meets classic Li-Fraumeni syndrome criteria: a proband with sarcoma diagnosed before 45 years, a first-degree relative with any cancer before 45 years, and a second-degree relative with any cancer before age 45 years or a sarcoma at any age [15]. For common diseases and moderate penetrance genes Bayesian analyses that require larger phenotype data sets are used [16]. Historically,* in vitro* studies were predominantly found in the published literature. However due to the rapid accumulation of rare variants, clinical laboratories such as Ambry Genetics are implementing validated internal functional studies such as splicing and homology-directed DNA break repair (HDR) assays that can be incorporated into variant classification algorithms.

## 3. Functional Lab

**Figure 2:**
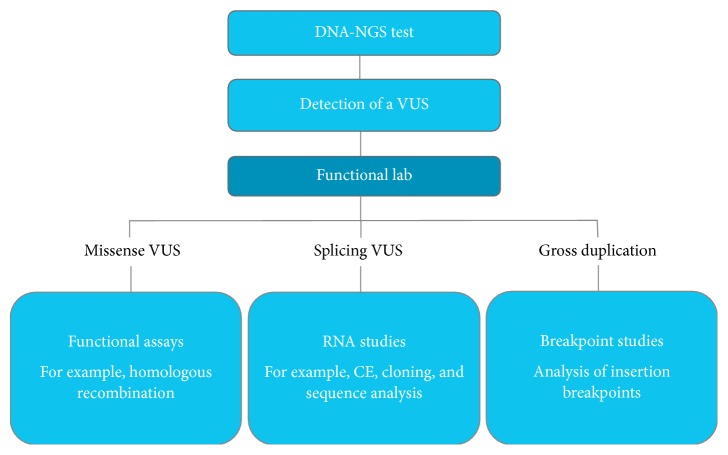
Workflow of a functional lab for the evaluation of VUS.

Many variants are classified as VUS because their functional impact either is poorly understood or has not yet been investigated. These variants include missense and splicing alterations in tumor suppressor genes that require loss of function to manifest a disease [7]. Clinical genomic laboratories have traditionally relied on evidence from published literature to establish the impact of a variant on gene expression or protein function [7]. There are several limitations to this approach, including publication bias, difficulties with promptly obtaining additional information about results and protocols, and lack of published evidence for a specific alteration. One potential solution is for clinical genomic laboratories to implement a “functional lab” that can generate assays with high sensitivity and specificity (>99%) and provide unbiased molecular evidence to elucidate the functional impact of a VUS (Figure 2). As an example of a convincingly validated assay, Guidugli and colleagues determined the sensitivity of their homology-directed DNA break repair (HDR) functional assay to be 100% (95% confidence interval (CI): 75.3%–100%) and the specificity to be 100% (95% CI: 81.5%–100%) [17].

### 3.1. RNA Studies for Splicing VUS

While some splicing variants, such as canonical ±1 or 2 splice sites, are often assumed to disrupt gene function by leading to the reduced expression of the abnormal allele due to nonsense-mediated decay (NMD) [18] or abnormal protein truncations [19], comprehensive evaluation of splicing alterations is essential for accurate clinical interpretation. For canonical splice site ±1 and 2 variants, one must also consider the possibility of an in-frame deletion/insertion, which could retain the critical regions of the protein and hence lead to a mild, neutral, or gain-of-function effect. In addition, variants that are predicted to impact splicing but that are not located at the canonical sites (±1 and 2) require additional strong evidence (see details in Section 2) to be classified as pathogenic or benign [7]. Bioinformatics software has been developed to predict putative splice sites [20]. In general, these* in silico* tools are more sensitive (~90–100%) than being specific (~60–80%) when predicting the impact of a variant on splicing [21, 22]. However, by nature* in silico* tools can only provide supporting evidence which restricts their use [7]. Consequently, data from RNA splicing assays, designed to provide quantitative and qualitative characterization of transcripts, are usually necessary to evaluate the pathogenicity of these variants. Since published RNA data is not available for every variant, clinical genomic laboratories can more accurately classify splicing alterations by implementing their own RNA protocols and assays to provide accurate classification of splicing alterations.

Reliability, in which an assay yields the same results in repeated trials, is a key issue when implementing mRNA assays in a clinical functional lab for evaluation of VUS. To improve reliability, the ENIGMA consortium conducted a multicenter investigation to compare mRNA splicing assay protocols used by its members [23]. The consortium provided several recommendations for best practices in clinical testing of splicing alterations, including the standardization of protocols and the use of analytically sensitive detection methods [23]. Of the detection methods evaluated, capillary electrophoresis (CE) was shown to yield the highest analytic sensitivity. However, a major limitation of CE is its inability to harvest and subsequently perform sequence analysis of the RT-PCR product. In order to perform sequence analysis and full characterization of alternatively spliced transcripts, the consortium concluded that cloning single PCR products into a vector system is a useful alternative for isolating single transcripts for sequencing, which improves sensitivity over band excision and sequencing alone. Even in cases that appear straightforward, the consortium recommends using* in vivo*,* in vitro*, and clinical analysis to predict with 99% likelihood that a variant is benign or pathogenic [23]. For example, although most canonical splice site variants are considered* a priori* to be at least likely pathogenic, the presence of naturally occurring alternative splicing that mimics a pathogenic alteration and results in a similar impact on splicing (e.g., exon skipping) needs to be carefully evaluated, as it may result in diminished pathogenicity. Care must be taken to determine whether a transcript is present in normal controls. As the functional lab obtains more data on each gene, a more accurate picture of splicing patterns will emerge, thereby leading to improved classification of splice site variations.

### 3.2. Functional Assays for Missense VUS

Missense alterations with no impact on splicing can be evaluated by utilizing wet lab assays or experimental structure data. While functional studies can be a powerful tool in support of pathogenicity, not all functional studies accurately predict impacts on gene or protein function. For this reason ACMG/AMP provides recommendations for assessing the validity of functional assays, in order to confirm that the functional assay accurately measures a function that leads to disease [7]. One must consider how closely the functional assay reflects the biological environment. This is important when deciding whether to test patient samples or to perform* in vitro* assays. It is important to consider the known biological functions of the protein, while also examining whether those functions actually contribute to tumorigenicity. For example, many functional assays have been developed to interrogate* BRCA1* VUS [24]. Some assays focus on the known DNA repair functions of BRCA1, such as the HDR assay [25, 26] and the radiation resistance assay [27]. Others examine BRCA1 localization [28, 29] and the ability of cells with* BRCA1* variants to generate Rad51 foci [30, 31] in the presence of DNA damage as surrogates for BRCA1 function. Additional assays focus on one functional component of BRCA1 instead of the full protein, including the transcription activation assay, which employs the C-terminal BRCT domains, and the ubiquitin ligase assay, which utilizes the N-terminal region [32–35]. These two assays are limited by their inability to account for effects of the entire protein, and others have noted that certain variants that lost ubiquitin ligase activity were not classified as pathogenic by genetic studies [36, 37]. Similarly, protein or peptide binding assays may resolve the ability of a variant to bind to a protein target* in vitro*, but these data should be incorporated into a multifactorial model that takes into account other functional* in vivo* data [38, 39]. In addition, validation data that assess the analytical performance of the assay and account for specimen integrity are important factors to consider when implementing functional assays in a functional clinical genomic laboratory and in using these results in classification of variants [7].

To investigate the effect of missense variants on BRCA1 function, Lu et al. tested 68 missense variants using an* in vitro* HDR assay [26]. The analysis showed that the HDR defective or partial defective missense variants from the BRCT domain are positioned either in the center of the structure or on the surface responsible for protein-protein interactions, while the HDR-WT variants from the BRCT domain were surface exposed or partially surface exposed variants [26]. This highlights the complexity of interpreting missense germline variants, indicating that an integrated approach, by compiling the results of functional assays, structure evaluation, and analysis of clinical parameters, should identify the most functionally and clinically relevant alterations.

### 3.3. Analysis of Insertion Breakpoints for Gross Duplications

Most gross deletions in high-risk cancer genes, larger than 3~5 megabases, fall within microarray reporting guidelines and are reported as deleterious [38, 39]; however, without breakpoint information gross duplications are mostly reported as VUS. While array comparative genomic hybridization (aCGH) is a method used in cancer research for the detection of gross chromosomal aberrations in cancer genes, it cannot accurately determine the exact genomic breakpoints of the amplification [40–43]. To map the exact insertion breakpoints, paired-end high throughput sequencing can be used. Gross genomic amplifications may occur as a tandem duplication within the cancer gene itself, resulting in a novel function, or as a nontandem duplication inserted in a novel location of the genome. Therefore, identifying the exact breakpoints of tandem duplications in high-risk cancer genes can lead to VUS being reclassified as likely pathogenic or likely benign.

**Figure 3:**
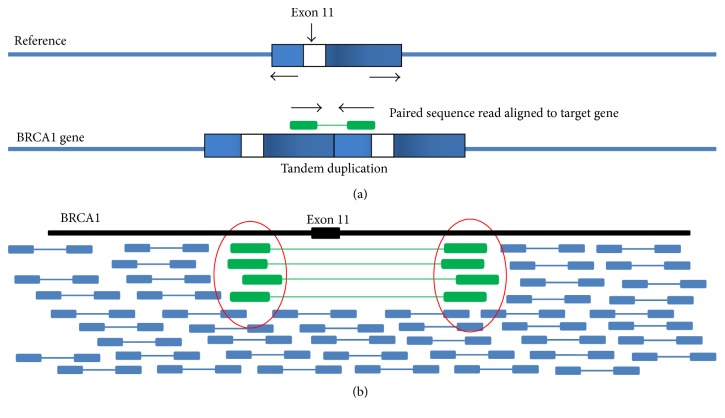
Identification of tandem duplication insertion breakpoints spanning* BRCA1* exon 11, using paired-end sequencing. (a) Mapped read pairs in the wrong orientation indicate a tandem duplication. (b) Ambry's breakpoint detection tools can identify clusters of read pairs with soft clipping which indicate rearrangement breakpoints.

To identify the exact breakpoints of tandem duplications, Ambry Genetics is currently utilizing the paired-end sequencing method to further characterize gross duplications. Probe sets are designed to capture the target regions with the suggested breakpoints identified by aCGH. Captured DNA is then sequenced by paired-end massively parallel sequencing and mapped to the human genome. The Ambry Genetics pipeline identifies read pairs that are in the wrong orientation, indicating a tandem duplication (Figure 3(a)). Clusters of read pairs with soft clipping that span breakpoints can indicate rearrangement breakpoints down to the exact coordinates (Figure 3(b)). As an example, an exon 11 duplication in* BRCA1* previously classified as VUS can be reclassified as likely pathogenic if the breakpoint is identified to cause a frameshift in the gene (Figure 3).

## 4. Computational Structural Analysis

Computational structural algorithms offer a unique solution for assessing a variant's impact on protein function in that they are faster than experimental studies and often use data from many scientific disciplines [44]. However, the quality of the information provided by computational analyses varies depending on the information source. For instance, primary sequence analyses using evolutionary tools can identify the likely impact of a variant. By comparing an altered human sequence to proteins with a similar primary sequence or related structural shape, the fitness of the variant can be predicted based on the variability of that position and other aspects such as the chemical similarity of the wild type and variant amino acids. Ambry Genetics relies on multiple tools, including the “Sorting Intolerant from Tolerant” (SIFT) and Polyphen2 programs [40]. We use the consensus of two programs, usually SIFT and Polyphen2 where applicable, and consider only concordant results as a line of evidence. If only one program is applicable such as Provean [45] with indels we incorporate predictions from the single program with conservative thresholds determined by analysis of our internal data. Alternatively, analyses of the secondary and tertiary structures of the protein increase the reliability of interpretation. The most reliable computational algorithms focus on biophysics methods which are more oriented towards direct simulation of the physical processes occurring in a protein [46–48]. In many regards, computational methods are the most diverse in the range of properties that they can quantify; however, they come at the expense of computational requirements and speed with which accurate properties can be derived. One of the most common and easily identified sources of disruption induced by a variant is the influence on protein stability [46, 47, 49–51]. Protein stability can be affected in multiple ways, such as misfolding or unfolding of the protein structure, which commonly results in either loss of function or premature degradation and haploinsufficiency. As an example, protein stability has been used by Karchin et al. to generate a predictive tool for the likelihood of the effect of an alteration in the breast cancer gene* BRCA2* [52]. There are other significant ways that variants exert their pathogenic effect which can be described through structure. For instance, a variant may not significantly affect the resting state structure of the protein but rather affect the movement of the protein in the course of its function. It may impact its binding with other target proteins or substrates or it may induce aggregation [47, 48]. Detailed understanding of biophysical principles illuminated through structure is crucial to evaluate and interpret the impact of alterations.

## 5. Tertiary and Quaternary Sequence of Breast Cancer Genes

The use of biophysical methods to predict the impact of a variant on a protein often requires the availability of structures for the target gene or benefits significantly from it. Among the 17 genes represented in the BreastNext Cancer panel, there are a total of 247 experimentally derived structures, tabulated per gene in the PDBs (Protein DataBank files) column of Table 2, using either Nuclear Magnetic Resonance Imaging (NMR) or X-ray crystallographic methods [5, 53]. The coverage described above corresponds to the total range of residues covered by all experimental measurements divided by the total length of the protein. While there are notable exceptions where no experimental structures have been determined, the majority of the genes have been partially and in the case of* TP53* completely elucidated experimentally. The structure of* TP53* is highly ordered throughout the protein, allowing for complete measurement of one low-energy form; however some proteins in this set such as* BRCA1* and* BRCA2* are composed of regions which have no characteristic fixed structure. The ordered regions within the structured protein, such as in the N (Really Interesting New Gene, i.e., RING, domain) and C terminus (BRCA1 C Terminus, i.e., BRCT, repeats) of* BRCA1*, offer higher quality means to define domain boundaries. These can be analyzed as a folded functional unit rather than through conservation techniques that are used in the Protein Families (Pfam) database [54] or using meta predictors such as InterPro [55]. The structural coverage for the genes in Table 2 does not take into account that long stretches of some proteins have little intrinsic globular structure, so the numbers can be seen as a very conservative estimate of the range of available residues covered. In addition, there remain some proteins, such as* ATM* or* NBN*, where no or low-resolution structures have been experimentally measured [5, 6]. For these systems, structural analysis incorporates the use of homology models built on the structures of known related proteins. This significantly increases the effective range of structural coverage and the insights available.

**Figure 4:**
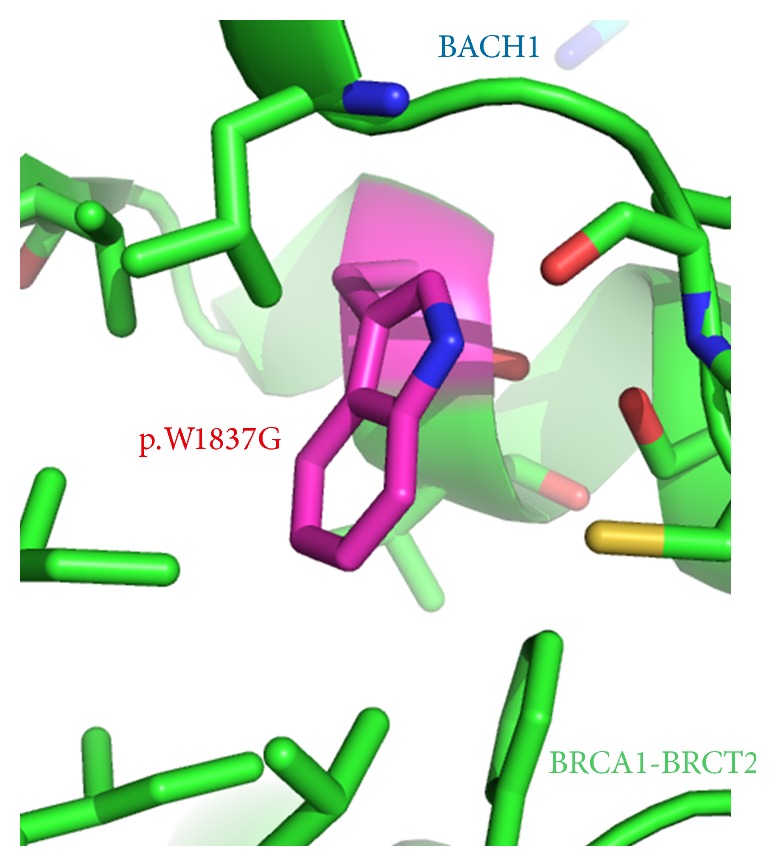
The structure of* BRCA1* p.Trp1837 (shown in magenta with sticks) in the BRCA-BRCT domain (PDB: 1T15 [6]). Nearby hydrophobic amino acids sidechains from residue 1837 are shown as sticks. Bound BACH1 peptide is shown as teal stick.

Disruption in the folding of a domain in a protein by a missense pathogenic variant is well known to result in a loss of function. The clinically observed alteration c.5509T>G (p.Trp1837Gly) (ClinVar: SCV000077040) represents a case where structural features explain the disruption of the BRCT repeat region in* BRCA1*. The C-terminal portion of* BRCA1* contains a pair of BRCT repeat domains, BRCT1 and BRCT2, which are described in atomic detail, including the arrangement of amino acids that make up these domains, by 26 different crystal structures [56–59]. The side chain of amino acid Trp 1837 (W1837; magenta stick) is buried in the core of the BRCT2 domain surrounded by hydrophobic amino acids (green sticks), while the backbone participates in a helix involved in binding BACH1 (Figure 4) [56]. The alteration W1837 to G1837 (W1837G) would result in the loss of the large stabilizing hydrophobic side chain and is anticipated to be very destabilizing. The instability introduced by this alteration has been quantitatively calculated by computational folding algorithms which indicate it to be very destabilizing [5]. Indeed,* E. coli* expressed with* in vitro* mutants of p.W1837G produce an unfolded protein that was present only in inclusion bodies which could not be refolded [60]. In another set of biochemical and cell-based transcriptional experiments, this alteration resulted in compromised proteolysis and phosphopeptide-binding [39, 58, 59, 61]. Together, these functional data support the qualitative and quantitative structural observation that the variant would create a very unfavorable cavity within this domain, thereby disrupting folding and protein function. This example demonstrates how detailed structural analysis on publically available data can facilitate the understanding and interpretation of alterations on the function of a protein and can be supported by both computational and experimental observations.

## 6. Conclusion

Although cancer genetic testing has traditionally been limited to highly penetrant and well-characterized susceptibility genes, the application of multigene panels using massively parallel sequencing is steadily becoming more common in genetic cancer risk assessment due to reduced costs and increased efficiency. Multigene panels, in turn, tend to result in the identification of more variants per individual, the clinical significance of which needs to be assessed using multiple lines of weighted evidence. We present an integrated approach for assessing variants observed on hereditary breast cancer panels and believe that this improves the clinical management of patients with personal and family histories of breast cancer due to more accurate variant classification. Comprehensive variant assessment programs that integrate multiple lines of evidence aimed at assessing a variant's impact on protein function, fitness, and pathogenicity facilitate high-quality and efficient variant classification, providing increased benefit and reliability for patients.

## Figures and Tables

**Table 1:** Classification scheme for high penetrance autosomal dominant breast cancer genes.

Class	Classification	Category	Criteria
5	Pathogenic	A 1 needed (stand-alone)	(i) Alterations resulting in premature truncation (e.g., reading frame shift, nonsense)
			(ii) Other ACMG-defined mutations (i.e., initiation codon or gross deletion)
			(iii) Strong segregation with disease (LOD >3 = >10 meioses)

5	Pathogenic	B 4 needed (strong)	(i) Confirmed *de novo *alteration in the setting of a new disease (appropriate phenotype) in the family (ii) Significant disease association in appropriately sized case-control study(ies)
			(iii) Being detected in individuals satisfying established diagnostic criteria for classic disease without a clear mutation
			(iv) Last nucleotide of exon
			(v) Good segregation with disease (LOD 1.5–3 = 5–9 meioses)
			(vi) Deficient protein function in appropriate functional assay(s)
			(vii) Functionally validated splicing mutation
			(viii) Well-characterized mutation at the same position
			(ix) Other strong data supporting pathogenic classification (e.g., structural)

4	Likely pathogenic	1 needed	(i) Alterations at the canonical donor/acceptor sites (± 1, 2) without another strong (B-level) evidence supporting pathogenicity

4	Likely pathogenic	C 4 needed (supportive)	(i) Rarity in general population databases (dbSNP, ESP, 1000 Genomes, ExAC)
			(ii) *In silico *models in agreement (deleterious) and/or completely conserved position in appropriate species
			(iii) Moderate segregation with disease (at least 3 informative meioses) for rare diseases
			(iv) Other data supporting pathogenic classification (e.g., structural)
		3 of B	
		2 of B and at least 1 of C	
		1 of B and at least 3 of C	

3	VUS	Insufficient or conflicting evidence	
		Gross duplications without strong evidence for pathogenic or benign	

3	Likely benign	D 1 needed (strong)	(i) Intronic alteration with no splicing impact by RT-PCR analysis or another splicing assay (ii) Other strong data supporting benign classification

3	Likely benign	E 2 needed (supportive)	(i) Cooccurrences with mutations in the same gene (phase unknown)
			(ii) Cooccurrences with mutations in other high penetrant genes that clearly explain a proband's phenotype
			(iii) Subpopulation frequency in support of benign classification
			(iv) Intact protein function observed in appropriate functional assay(s)
			(v) *In silico *models in agreement (benign)
			(vi) Not segregating with disease in family study (genes with incomplete penetrance)
			(vii) No disease association in small case-control study
			(viii) Other data supporting benign classification

1	Benign	F 1 needed (stand-alone)	(i) General population or subpopulation frequency is too high to be a pathogenic mutation based on disease/syndrome prevalence and penetrance
			(ii) Not segregating with disease in family study (genes with complete penetrance)
			(iii) Internal frequency is too high to be a pathogenic mutation based on disease/syndrome prevalence and penetrance
			(iv) Being seen *in trans *with a mutation or in homozygous state in individuals without severe disease for that gene
			(v) No disease association in appropriately sized case-control study(ies)
		1 of D and at least 2 of E	
		2 or more of D	
		>3 of E w/o conflicting data	
		>4 of E w/conflicting data	

The variant classification scheme is not intended for the interpretation of alterations complicated by epigenetic factors including genetic modifiers, multifactorial disease, or low-risk disease association alleles and may be limited in the interpretation of alterations confounded by incomplete penetrance, variable expressivity, phenocopies, and triallelic or oligogenic inheritance.

**Table 2:** Experimental structures of genes linked to breast cancer^*∗*^.

Gene	Length	PDBs	Coverage (%)
ATM	3056	0	0.0
BARD1	777	5	42.1
BRCA1	1863	27	17.6
BRCA2	3418	2	1.6
BRIP1	1249	3	1.9
CDH1	882	12	26.2
CHEK2	543	38	86.4
MRE11A	708	1	58.1
MUTYH	549	2	77.3
NBN	754	0	0.0
NF1	2839	6	22.1
PALB2	1186	2	29.7
PTEN	403	6	92.8
RAD50	1312	0	0.0
RAD51C	376	0	0.0
RAD51D	328	1	25.3
TP53	393	142	100.0

^*∗*^Gene lengths and coverage are tabulated from the Universal Protein Resource (Uniprot) [4] and the Research Collaboratory for Structural Bioinformatics (RCSB) [5] databases. The list of genes is taken from the BreastNext panel.
